# Genomic prediction of the performance of tropical doubled haploid maize lines under artificial *Striga hermonthica* (Del.) Benth. infestation

**DOI:** 10.1093/g3journal/jkae186

**Published:** 2024-08-12

**Authors:** Joan J C Kimutai, Dan Makumbi, Juan Burgueño, Paulino Pérez-Rodríguez, Jose Crossa, Manje Gowda, Abebe Menkir, Angela Pacheco, Beatrice E Ifie, Pangirayi Tongoona, Eric Y Danquah, Boddupalli M Prasanna

**Affiliations:** Global Maize Program, International Maize and Wheat Improvement Center (CIMMYT), P.O. Box 1041–00621, Nairobi, Kenya; West Africa Centre for Crop Improvement (WACCI), University of Ghana, PMB 30 Legon, Accra, Ghana; Global Maize Program, International Maize and Wheat Improvement Center (CIMMYT), P.O. Box 1041–00621, Nairobi, Kenya; Biometrics and Statistics Unit, CIMMYT, Apdo. Postal 6–641, 06600 Mexico DF, Mexico; Socioeconomía, Estadística e Informática, Colegio de Postgraduados, Edo. de México 56230, Montecillos, Mexico; Biometrics and Statistics Unit, CIMMYT, Apdo. Postal 6–641, 06600 Mexico DF, Mexico; Socioeconomía, Estadística e Informática, Colegio de Postgraduados, Edo. de México 56230, Montecillos, Mexico; Global Maize Program, International Maize and Wheat Improvement Center (CIMMYT), P.O. Box 1041–00621, Nairobi, Kenya; International Institute of Tropical Agriculture (IITA), Oyo Road, PMB 5320, Ibadan, 200001, Nigeria; Biometrics and Statistics Unit, CIMMYT, Apdo. Postal 6–641, 06600 Mexico DF, Mexico; West Africa Centre for Crop Improvement (WACCI), University of Ghana, PMB 30 Legon, Accra, Ghana; Institute of Biological, Environmental & Rural Sciences (IBERS), Aberystwyth University, Aberystwyth, SY23 3EE Wales, UK; West Africa Centre for Crop Improvement (WACCI), University of Ghana, PMB 30 Legon, Accra, Ghana; West Africa Centre for Crop Improvement (WACCI), University of Ghana, PMB 30 Legon, Accra, Ghana; Global Maize Program, International Maize and Wheat Improvement Center (CIMMYT), P.O. Box 1041–00621, Nairobi, Kenya

**Keywords:** *Striga*, maize breeding, genomic prediction, doubled haploid, sparse phenotyping

## Abstract

*Striga hermonthica* (Del.) Benth., a parasitic weed, causes substantial yield losses in maize production in sub-Saharan Africa. Breeding for *Striga* resistance in maize is constrained by limited genetic diversity for *Striga* resistance within the elite germplasm and phenotyping capacity under artificial *Striga* infestation. Genomics-enabled approaches have the potential to accelerate identification of *Striga* resistant lines for hybrid development. The objectives of this study were to evaluate the accuracy of genomic selection for traits associated with *Striga* resistance and grain yield (GY) and to predict genetic values of tested and untested doubled haploid maize lines. We genotyped 606 doubled haploid lines with 8,439 rAmpSeq markers. A training set of 116 doubled haploid lines crossed to 2 testers was phenotyped under artificial *Striga* infestation at 3 locations in Kenya. Heritability for *Striga* resistance parameters ranged from 0.38–0.65 while that for GY was 0.54. The prediction accuracies for *Striga* resistance-associated traits across locations, as determined by cross-validation (CV) were 0.24–0.53 for CV0 and from 0.20 to 0.37 for CV2. For GY, the prediction accuracies were 0.59 and 0.56 for CV0 and CV2, respectively. The results revealed 300 doubled haploid lines with desirable genomic estimated breeding values for reduced number of emerged *Striga* plants (STR) at 8, 10, and 12 weeks after planting. The genomic estimated breeding values of doubled haploid lines for *Striga* resistance-associated traits in the training and testing sets were similar in magnitude. These results highlight the potential application of genomic selection in breeding for *Striga* resistance in maize. The integration of genomic-assisted strategies and doubled haploid technology for line development coupled with forward breeding for major adaptive traits will enhance genetic gains in breeding for *Striga* resistance in maize.

## Introduction

*Striga hermonthica* (Del.) Benth. is a parasitic weed that affects maize (*Zea mays* L.) production in sub-Saharan Africa (SSA). *Striga* spp. has a wide geographical distribution and affects up to 60% of the arable land in the region (Mbuvi *et al.* 2017). The weed adversely affects maize production in SSA causing yield losses ranging from 20–100% (Ransom *et al.* 1990; Berner *et al.* 1996; Khan *et al*. 2006; Ejeta 2007a, 2007b). *Striga* depends entirely on its host for growth and survival. Under favorable growing conditions, *Striga* seeds break dormancy in response to germination stimulants (Strigolactones) produced by the host. A germinated *Striga* plant then establishes vascular connections with the host's roots via the haustoria through which it draws nutrients and water resulting in stunted growth, chlorosis, impaired photosynthesis, reduced maize biomass, and yield loss (Gurney *et al.* 1995; Spallek *et al.* 2013).

Several control strategies have been proposed to reduce the burden of *Striga* for farmers in SSA. These include crop rotation (Oswald and Ransom 2001), intercropping (Khan *et al.* 2002), push–pull technology (Khan *et al.* 2008), host plant resistance (Menkir *et al.* 2007; Rich and Ejeta 2008), herbicide resistant maize (Makumbi *et al.* 2015), and integrated pest management (Khan *et al.* 2016; Kanampiu *et al*. 2018). Host plant resistance is one of the most promising approaches for *Striga* control as the technology is embedded in the seed. Host plant resistance, coupled with other control approaches, is considered an important *Striga* control strategy for smallholder farmers due to its ease of deployment and adoption (Mwangangi *et al.* 2021).

Breeding for *Striga* resistance is hampered by the limited sources of resistance within elite maize germplasm, complex genetics of resistance, complicated host–parasite relationship (Amusan *et al.* 2008), and limited phenotyping capacity. Phenotyping for *Striga* resistance or tolerance requires uniform artificial *Striga* infestation that exposes maize seedlings to a large number of *Striga* seeds to prevent escape (Kim 1996; Kling *et al.* 1999). Although the artificial *Striga* infestation technique has been successful, breeders are limited by lack of large experimental fields that can solely be dedicated for artificial screening. This can slow progress in identifying resistant inbred lines and hybrids as a limited number of genotypes can be screened at a time. Despite these challenges, significant progress has been made in developing and deploying *Striga* resistant maize varieties in West Africa by the International Institute of Tropical Agriculture (IITA, https://www.iita.org) and its partners over the years (Kim *et al.* 1994; Badu-Apraku *et al.* 2007; Menkir and Kling 2007; Menkir *et al.* 2012; Menkir and Meseka 2019). A study by Menkir *et al*. (2007) showed that the key traits for *Striga* resistance breeding namely grain yield, *Striga* damage rating, and *Striga* counts are conditioned by many genes with small effects. Recurrent selection studies have shown improvements in *Striga* resistance related traits in maize in West Africa (Menkir and Kling 2007; Badu-Apraku *et al.* 2009; Badu-Apraku 2010). Recent studies reported genetic gains of 93.7 kg ha^−1^ year^−1^ (Menkir and Meseka 2019) and 101 kg ha^−1^ year^−1^ (Badu-Apraku *et al.* 2020a) for grain yield under *Striga* infestation. These gains were attributed to significant gains in the reduced number of emerged *Striga* plants and less *Striga* damage. Menkir and Meseka (2019) reported gains of −6.7% and −5.5% year^−1^ for number of emerged *Striga* plants at 8 and 10 weeks after planting (WAP), respectively. The reported genetic gains are attributed to the use of effective screening protocols (Kim 1994; Kim and Adetimirin 2001), and better understanding of the genetics of *Striga* resistance (Kim 1994; Yallou *et al.* 2009; Badu-Apraku *et al.* 2013).

The genetic gains reported in breeding for *Striga* resistance at IITA have been achieved through development of inbred lines using conventional pedigree breeding method and backcrossing. In addition, recurrent selection has been used to accumulate desirable alleles for traits associated with resistance to *Striga* (Badu-Apraku *et al.* 2007; Menkir and Kling 2007). Developing near-homozygous inbred lines in 6–8 generations through the pedigree method could slow the rate of genetic gain in breeding for resistance to *Striga* in maize. The use of the doubled haploid (DH) technology in maize through which completely homozygous lines can be developed within 13–14 months could significantly reduce the breeding cycle time, and accelerate population and variety development (Bernardo 2009; Chaikam *et al.* 2019). Application of DH technology for line development for SSA has been implemented at a large scale at CIMMYT since 2012 (Prasanna *et al.* 2012; Chaikam *et al.* 2019).

The application of marker assisted selection along with conventional breeding and DH technology can speed up the identification of *Striga* resistant germplasm. Several quantitative trait loci related to *Striga* resistance have been reported (Badu-Apraku *et al.* 2020b, 2020c, 2023). Genome-wide association studies have identified significant single nucleotide polymorphisms (SNPs) associated with number of emerged *Striga* plants and *Striga* damage rating in tropical maize (Adewale *et al.* 2020; Stanley *et al.* 2021; Gowda *et al.* 2021; Okunlola *et al.* 2023). Accelerated line and variety development can also be achieved through the incorporation of genomic selection (GS) in a breeding program. The use of DH lines in combination with genomic prediction/selection methods can accelerate genetic improvement in crop plants (Heffner *et al.* 2010; Song *et al.* 2017; Cerrudo *et al.* 2018).

Genomic selection is an approach for improving complex quantitative traits. Genomic selection (Meuwissen *et al.* 2001) and genomic prediction of complex traits (de los Campos *et al.* 2009; Crossa *et al.* 2010; Pérez-Rodríguez *et al.* 2012) target breeding value estimates which include the parental average and a deviation resulting from Mendelian sampling (Heffner *et al.* 2009; Crossa *et al.* 2017). Genomic prediction has been used to estimate additive as well as nonadditive effects of lines (Crossa *et al.* 2017; Bonnett *et al.* 2022). Estimation of additive gene effects allows for selection in early generations such as F_2_ (Crossa *et al*. 2017). Genomic prediction accounts for Mendelian segregation and considers the realized covariances based on dense molecular markers that span the genome (Pérez-Rodríguez *et al.* 2012). With both marker and phenotypic data, the genetic values of genotypes evaluated in single and across environments are estimated using genomic prediction through genotype by environment (G × E) interaction analyses. Research on crop and animal breeding has shown that prediction accuracy in selection for complex traits using pedigree information can significantly be improved through genomic selection with different models (Crossa *et al.* 2022).

Multiple genomic prediction models including parametric and nonparametric statistical and computational models that account for both genetic and nongenetic effects have been developed to estimate genomic breeding values (GEBVs) (Crossa *et al.* 2017). Additionally, linear and nonlinear kernels that are based on genomic relationship matrices have been reported to be better than the conventional methods (Crossa *et al.* 2022). Nonlinear genomic kernels such as the reaction norm model can account for epistatic effects between markers and incorporate large-scale environmental data (enviromics) and G × E analyses for improved prediction accuracy (Jarquín *et al.* 2014). The prediction accuracy of the model is assessed through cross-validation after which an appropriate model is used to predict the performance of untested genotypes by estimating their genomic breeding values. The candidate lines are therefore selected based on GEBVs generated from the marker and phenotype information of the training population (Crossa *et al.* 2017). Only genotypes with the best GEBVs are selected and advanced depending on the trait. Genomic selection can thus accelerate breeding by reducing the duration of line and variety development, while also reducing phenotyping costs in crops like maize (Crossa *et al.* 2013; Edriss *et al.* 2017; Beyene *et al.* 2021; Butoto *et al.* 2022), and in other crops (Pérez-Rodríguez *et al.* 2012; Iwata *et al.* 2015; Velazco *et al.* 2019).

The use of genomic selection in breeding programs focusing on improving *Striga* resistance for increased genetic gains in grain yield under artificial *Striga* infestation could provide an option to overcome the challenge of limited and costly phenotyping. The International Maize and Wheat Improvement Center (CIMMYT, https://www.cimmyt.org) has developed several DH lines using *Striga* resistant maize germplasm from IITA. This germplasm could provide insights on the application of genomic selection for the incorporation of *Striga* resistance in mid-altitude maize germplasm in Eastern and Southern Africa where *S. hermonthica* still presents a major challenge. The objectives of this study were to (1) assess the efficiency of genomic prediction for *Striga* resistance-associated traits and grain yield using the reaction norm model, and (2) predict the genetic values of field tested and untested DH lines.

## Materials and methods

### Genetic material

This study utilized 606 DH lines developed by CIMMYT at the Maize DH Facility in Kiboko, Kenya (Supplementary Table 1). The DH lines were developed from induction of F_2_ and BC_1_F_2_ populations formed by crossing *Striga* resistant donor lines from IITA with elite mid-altitude tropical maize lines developed by CIMMYT. The *Striga* resistance donor lines from IITA include TZSTR182, TZSTR184, TZISTR1156, TZISTR1158, and TZSTR167. Line TZSTR167 was derived from a yellow composite (TZLCOMP1.Y), whereas lines TZSTR182, TZSTR184, TZISTR1156, and TZSTR1158 were derived from bi-parental crosses of white inbred lines derived from a Striga resistant synthetic (ACRSYN-W) and a composite (TZLCOMPIC4). The elite CIMMYT lines (CML521, CML522, and CML543) used for crossing had varying levels of drought tolerance and/or herbicide (imazaypr) resistance. Some F_1_ crosses were advanced to F_2_ while others were planted alongside either the IITA donor lines or the adapted CIMMYT lines and crossed to form BC_1_F_1_. The BC_1_F_1_ were selfed to form BC_1_F_2_ populations which were then submitted for DH induction. There were 171 and 435 DH lines developed from F_2_ and BC_1_F_2_ populations, respectively. Of the 606 DH lines, 116 lines derived using CML522 (a drought tolerant and herbicide resistant line) as a parent were selected to serve as the training population (TRN) and crossed to 2 inbred line testers from IITA to form 232 testcross hybrids.

### Experimental design, test locations, and artificial *Striga* infestation

The 232 testcross (TC) hybrids were part of 351 TC hybrids that were developed from new DH lines and were tested in 2 trials. Trial 1 had 180 entries while Trial 2 had 171 entries. Each trial included 116 TC hybrids from the TRN set. Only 232 TC hybrids were used for this study as only 116 lines had both genotypic and phenotypic data. Trial 1 included 2 internal genetic gain checks and 6 commercial checks while Trial 2 had 2 internal genetic gain checks and 7 commercial checks. The experimental design was 4 × 47 and 4 × 45 alpha-lattice with 2 replications for Trials 1 and 2, respectively. Each experimental unit consisted of one 4 m row spaced 0.75 m apart and 0.20 m space between plants, giving a plant population density of approximately 66,666 plants ha^−1^ at all locations. The hybrids were evaluated in field trials under artificial *Striga* infestation at the Kenya Agricultural and Livestock Research Organization (KALRO) research stations at Kibos (0°2′S, 34°48E, 1,193 masl) and Alupe (0°30′N, 34°7E, 1,250 masl), and at Siaya ATC (03°10′N, 34°17E, 1,288 masl) in 2020. The soil types are classified as Eutric Cambisol, Orthic Ferralsol, and Plinthic Ferralsol at Kibos, Alupe, and Siaya ATC, respectively. All locations have a bimodal rainfall distribution (March–July and September–November), with most of the rain falling between March and July. The fields used for artificial *Striga* infestation at the research stations had been previously used for imazapyr herbicide studies (Kanampiu *et al.* 2002, 2018; Makumbi *et al.* 2015), whose residual toxicity (Alister and Kogan 2005) kills *Striga* seed in the soil.

To obtain uniform exposure to *Striga* for each genotype, artificial *Striga* infestation was used. *Striga* seed was collected from infested maize fields in the *Striga* infested belt of western Kenya (Gethi *et al.* 2005). *Striga* inoculum was prepared by thoroughly mixing 10 g of *Striga* seeds, with 5 kg of sand. The *Striga* seed-sand inoculum (20 g) was applied to each planting hole at a depth of 7–10 cm using a calibrated spoon that delivered up to ∼3,000 *Striga* seeds to ensure uniform *Striga* infestation in the trials (Makumbi *et al.* 2015). The *Striga* seed-sand inoculum was placed directly at the bottom of the planting hole for uniform exposure of the maize plants to *Striga* from the onset of germination. Di-ammonium phosphate (DAP, 18:46:0) fertilizer was applied at half the recommended rate (30 kg ha^−1^) at planting to enhance plant establishment but avoid suppressing *Striga* germination. Half dose (30 kg ha^−1^) of calcium ammonium nitrate (CAN, 26%) fertilizer was used for topdressing at 4 WAP. Standard agronomic and cultural practices were performed as recommended for each location. Hand weeding was carried out to eliminate all weeds except *Striga* plants.

### Data collection

Data were recorded on the number of emerged *Striga* plants (STR), *Striga* damage rating (SDR), and ear weight. The number of emerged *Striga* plants per plot was recorded within 15 cm of either side of the row at 8, 10, and 12 WAP. The SDR was recorded at 10 (SDR1) and 12 WAP (SDR2) using a 1–9 rating scale where 1 refers to a healthy plant with no visible symptoms of *Striga* damage (resistant) and 9 is highly susceptible to *Striga* with totally scorched leaves, absent ears, and untimely death of the host plant (Kim 1991; Kim *et al.* 2002). The area under *Striga* number progress curve (AUSNPC) was computed from the 3 STR plant counts (8, 10, and 10 WAP) following the formula for calculating the area under disease progress curve (AUDPC) (Shaner and Finney 1977) as:


$$\mathrm{AUSNPC}=\overset{n}{\underset{i=1}{\sum }}\;\left(\frac{y_{i}+y_{i-1}}{2}\right)(t_{i}-t_{i-1}),$$

where $y_{i}$ is the number of *Striga* plants at the ith observation, $t_{i}\;$ is the time point in days after planting at the *i*th observation, and *n* is the total number of observations.

Finally, grain yield expressed in tons per hectare (t ha^−1^) was computed based on ear weight per plot, assuming 80% shelling percentage and adjusted to 12.5% grain moisture content.

### Genotypic data

Leaf samples of the 606 DH inbred lines were collected 3 WAP and shipped to Intertek laboratories in Sweden for DNA extraction. The DNA samples were then forwarded to the Institute for Genomic Diversity, Cornell University (Ithaca, NY, USA) for genotyping with repetitive amplicon sequences (rAmpSeq markers). A genome indexing approach was used for designing primers using the conserved regions of the genome. The repeat amplicons were then multiplexed for genotyping as described by Buckler *et al*. (2016). The rAmpSeq protocol is a simple cost-effective sequencing technology which uses targeted amplicon sequencing approach and gene specific primers to amplify targeted regions of interest. The DNA library was constructed, mapped to B73 maize reference genome (version 3) and each unique sequence tag was regarded as a dominant marker. The dominant markers were saved in present–absent variant format where one (1) and zero (0) denoted present or absent, respectively. For the 606 DH lines, a total of 8,439 sequence tags were called. The marker quality control (QC) process which involved the exclusion of monomorphic and uninformative markers, markers with minor allele frequencies < 0.05 and those whose variances were equal to 0 was carried out in R Software (R Core Team 2022). After QC, 5,380 high quality rAmpSeq markers were selected for use in genomic prediction.

### Statistical analyses

#### Analysis of variance

*Striga* count data were tested for normality using the Shapiro–Wilk test before conducting analysis of variance. Analysis of individual trials was carried out using META-R (Alvarado *et al.* 2020). The best linear unbiased estimates (BLUEs) and the best linear unbiased predictions (BLUPs) were computed by a linear mixed model in which genotype effect was considered as fixed and random, respectively. The BLUEs were used for the genomic prediction model as input data while the random models were used to evaluate quality of individual trials. All other effects in the model were considered random. The linear mixed model used for single site analysis is as follows:


$$y_{ijk}=\mu +G_{i}+R_{j}+B_{k}(R_{j})+\varepsilon_{ijk},$$

where $y_{ijk}$ is the response variable; *μ* is an intercept; $G_{i}$ is the effect of the *i*th genotype; $\;R_{j}$ is the effect of *j*th replicate; $B_{k}(R_{j})$ is the effect of the *k*th block within the *j*th replicate; while $\varepsilon_{ijk}$ is the experimental error associated with the *i*th genotype, *j*th replicate, and *k*th block. We assumed $\varepsilon ∼\mathrm{NIID}(0,\;\sigma_{\varepsilon }^{2}$), where NIID is normal independent and identically distributed random variables, $\sigma_{\varepsilon }^{2}$ is the associated variance parameter.

After individual analysis, data were analyzed combined across locations with a linear mixed model using ASReml-R version 4.2 (Butler *et al.* 2009). From this point, moving forward, the environment is synonymous with location. The linear mixed model fitted for the combined analysis was:


$$y_{ijkl}=\mu +G_{i}+E_{j}+R_{k}(E_{j})+B_{l}(ER{)}_{\;jk}+GE_{ij}+\varepsilon_{ijkl},$$

where $y_{ijkl}\;$ is the response variable; *μ* is an intercept; $G_{i}$ is the effect of the *i*th genotype; $E_{j}$ is the effect of the *j*th environment; $R_{k}(E_{j})$ is the effect of the *k*th replicate in the *j*th environment; $B_{l}(ER{)}_{\;jk}$ is the effect of the *l*th block within the *k*th replicate at the *j*th environment; $GE_{ij}$ is the effect of the interaction between the *i*th genotype and the *j*th environment; while $\varepsilon_{ijkl}$ is the experimental error associated with the *i*th genotype, *j*th environment, *k*th replicate, and *l*th block where the error term is assumed to be normally, identical, and independently distributed (NIID) with mean 0 and homoscedastic variance $\sigma_{\varepsilon }^{2}$. All effects except *μ* and *E_j_* were considered random effects.

Broad-sense heritability was estimated for individual and combined environments according to Hallauer *et al*. (2010). At individual environments, heritability was computed as:


$$H_{a}^{2}=\frac{\sigma_{G}^{2}}{\left[\sigma_{G}^{2}+\frac{\sigma_{\varepsilon }^{2}}{R}\right]},$$

where $H_{a}^{2}$ is the broad-sense heritability for individual environments, $\sigma_{G}^{2}$ is the genotypic variance, $\sigma_{\varepsilon }^{2}$ is the variance associated to the error, and R is the number of replications. The heritability across environments was computed as:


$$H_{b}^{2}=\frac{\sigma_{G}^{2}}{\left[\sigma_{G}^{2}+\frac{\sigma_{GE}^{2}}{E}+\frac{\sigma_{\varepsilon }^{2}}{E\times R}\right]},$$

where $H_{b}^{2}$ is the broad-sense heritability for combined environments, $\;\sigma_{G}^{2}$ is the genotypic variance, $\sigma_{GE}^{2}$ is the variance of the interaction between the genotype and the environment, *E* is the number of environments, *R* is the number of replicates, and the $\sigma_{\varepsilon }^{2}$ is the residual variance. BLUPs obtained from the combined phenotypic analysis were used to calculate Pearson's correlation coefficients among the different traits.

#### Genomic prediction

We computed a genomic relationship matrix (GRM) according to Lopez-Cruz *et al*. (2015) for use in subsequent analysis. The GRM was computed as; G=M/p, where M is the matrix of markers centered and standardized by column (mean 0 and variance 1 by marker) and *p* is the number of markers. The objective of genomic prediction was to estimate the number of emerged *Striga* plants, *Striga* damage rating, AUSNPC, and grain yield for lines not evaluated in the field. Given that some of the genotyped lines were evaluated at 3 locations (Kibos, Alupe, and Siaya), we employed the reaction norm model proposed by Jarquín *et al*. (2014) to predict GEBVs considering the environments, markers and the interaction between genotypes and environments. The BLUEs obtained from phenotypic analysis were used for genomic prediction. The equation for the reaction norm model is:


$$y=Z_{E}\beta_{E}+Z_{g}g+u+e,$$

where y is the BLUEs of the response vector (number of emerged *Striga* plants, *Striga* damage rating, AUSNPC, or grain yield), $Z_{E}$ is a design matrix for environments (locations), $\beta_{E}$ is the vector effect of the environments, $\beta_{E}\;∼\;\mathrm{MN}(0,\sigma_{E}^{2}I)$, where MN is multivariate normal distribution, **0** is a vector or zeros, $\sigma_{E}^{2}$ is the variance parameter associated with environments, and I is the identity matrix; $Z_{g}$ is a matrix that connects phenotypes with genotypes, and g is the vector of random effects of genotypes. We assumed $g∼\mathrm{MN}(0,\;\sigma_{g}^{2}G)$ with $\sigma_{g}^{2}$ the variance associated to the genotypes, G is a genomic relationship matrix (Lopez-Cruz *et al.* 2015); u represents the interaction, we assumed $u∼\mathrm{MN}(0,\sigma_{g\times E}^{2}Z_{g}{GZ}_{g}^{t}\#\;Z_{E}Z_{E}^{t})$, with $\sigma_{g\times E}^{2}$ the variance parameter associated to the interaction and # representing the element-wise product of 2 matrices. Finally, e represents the error, we assumed $e∼\mathrm{MN}(0,\sigma_{e}^{2}I)$, with $\sigma_{e}^{2}$ the variance associated to the error. Furthermore, we also assumed that $\beta_{E},\;g,\;u$, and e are distributed independently. In this study, no environmental variables were considered and therefore the environmental effect corresponds to a dummy location effect. The training set (TRN) consisted of phenotypic data of 116 DH lines evaluated in 232 testcrosses at Kibos, Alupe, and Siaya under artificial *Striga* infestation while the testing set (TST) consisted of the 490 DH lines not evaluated in the field.

#### Cross-validation

Two cross-validations schemes were used to determine the prediction accuracy of the reaction norm model. Using the reaction norm model (Jarquín *et al.* 2014), 2 main prediction scenarios were considered: cross-validation 1 (CV1) and cross-validation 2 (CV2) (Burgueño *et al.* 2012). The CV1 was used to predict the performance of new lines that have not been field screened under artificial *Striga* infestation while CV2 sought to predict the genetic value of the lines in locations in which they have not been tested but were tested in other environments. For the computation of both CV1 and CV2 correlation values, 20% of the lines were considered as the testing set while the remaining 80% were used to train the model in 50-fold cross-validations. The training data set was used to train the model while testing set was used to estimate the model prediction accuracy measured by the Pearson's correlation coefficient between observed and predicted values. For each of the 50 random partitions, prediction accuracy was computed within and across environments (locations) for all traits. The reaction norm model was fitted using the BGLR package in R (Pérez-Rodríguez and de los Campos 2014). Inferences were based on 30,000 iterations with a thin of 10, obtained after discarding the first 15,000 iterations that were taken as burn-in.

To evaluate the prediction accuracy in each environment, a third form of cross-validation (CV0) involving use of phenotypic data from 2 environments to estimate the prediction accuracy of the model in estimating the performance of lines in the third environment was carried out. The prediction accuracy for each environment was estimated when the phenotypic data in that specific environment was treated as missing values (the testing set) using BGLR (Pérez-Rodríguez and de los Campos 2014).

## Results

### Analysis of variance and testcross performance

**Fig. 1.**
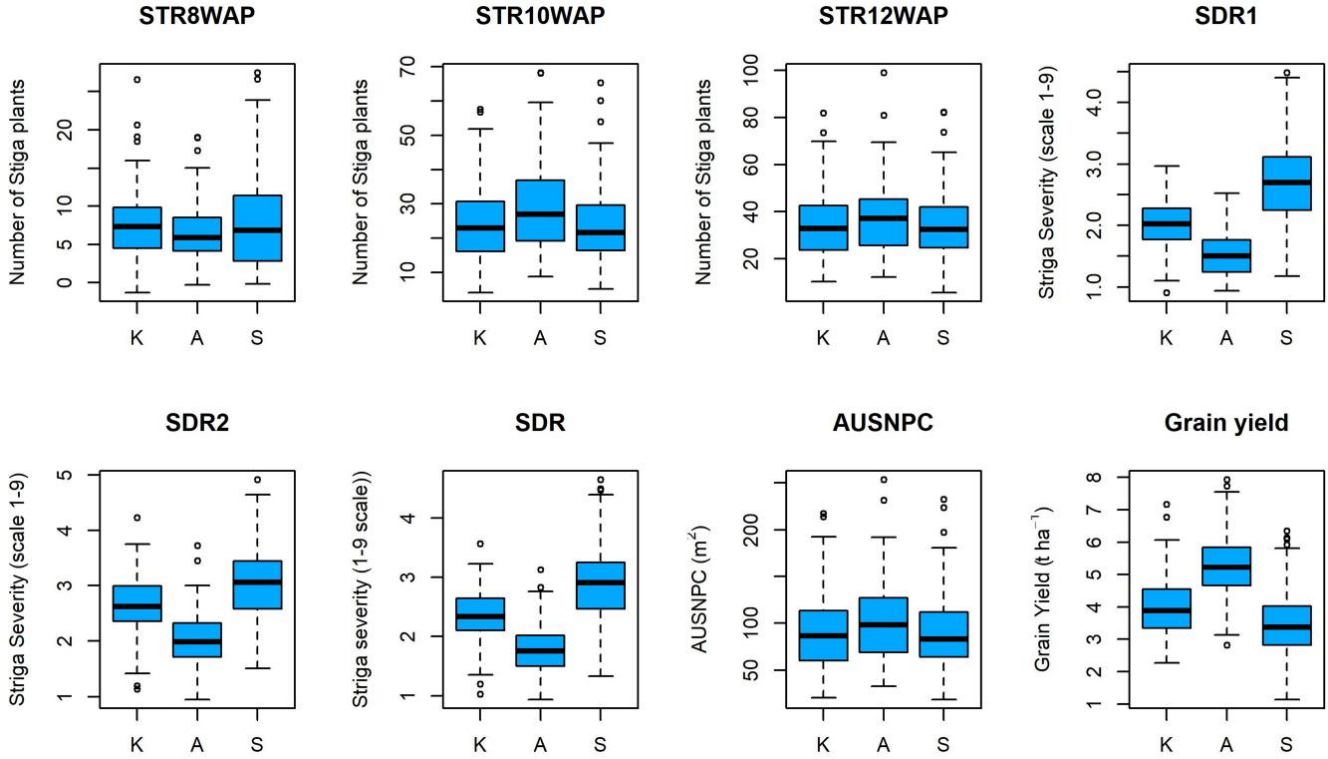
Boxplots of *Striga* resistance parameters and grain yield at the 3 trial locations in Kenya (K, Kibos; A, Alupe; S, Siaya) in 2020. STR8WAP, emerged *Striga* plants 8 weeks after planting (WAP); STR10WAP, emerged *Striga* plants 10 WAP; STR12WAP, emerged *Striga* plants 12 WAP; SDR1 and 2, *Striga* damage rating at 10 and 12 WAP, respectively; SDR, average *Striga* damage rating; AUSNPC, area under *Striga* number progress curve (m^2^).

In this study, we used 606 new DH lines of which 116 were crossed to 2 testers to generate 232 testcross hybrids that were phenotyped under artificial *Striga* infested conditions at 3 locations in Kenya. Analysis of variance at individual locations showed significant variation among hybrids for all traits measured (Table 1). The magnitude of genetic variance for number of emerged *Striga* plants at 10 WAP (STR10WAP) and 12 WAP (STR12WAP) was 8.2 and 16.5 times greater than that for emerged *Striga* plants at 8 WAP (STR8WAP), respectively. Broad-sense heritability was low to moderate for *Striga* resistance parameters (0.23–0.54) and moderate for grain yield (0.31–0.53). Broad-sense heritability for the *Striga* resistance parameters was lower at Siaya compared to the other 2 locations. The mean number of emerged *Striga* plants at 8 WAP was the lowest at Alupe (7), but the same location recorded the highest mean number of emerged *Striga* plants at 10 WAP and 12 WAP (Fig. 1). The *Striga* damage rating (SDR), at 10 WAP, 12 WAP, and the average SDR were highest at Siaya and lowest at Alupe (Fig. 1). The AUSNPC was lowest at Kibos and Siaya (190 m^2^). Mean grain yield was highest at Alupe (5.3 t ha^−1^) and lowest at Siaya (3.3 t ha^−1^).

**Table 1.** Variance component estimates and heritability for different *Striga* resistance parameters and grain yield at 3 locations under artificial *Striga* infestation in 2020.

Trait	Kibos			Alupe			Siaya		
	${\hat{\sigma }}_{G}^{2}$	${\hat{\sigma }}_{\varepsilon }^{2}$	$H_{a}^{2}$	${\hat{\sigma }}_{G}^{2}$	${\hat{\sigma }}_{\varepsilon }^{2}$	$H_{a}^{2}$	${\hat{\sigma }}_{G}^{2}$	${\hat{\sigma }}_{\varepsilon }^{2}$	$H_{a}^{2}$
STR8WAP	16.75*^b^*	63.51	0.35	25.20*^b^*	77.05	0.40	16.23*^b^*	75.76	0.30
STR10WAP	136.66*^b^*	334.54	0.45	133.56*^b^*	325.07	0.45	44.14*^a^*	303.14	0.23
STR12WAP	275.95*^b^*	632.55	0.47	189.14*^b^*	408.31	0.48	194.70*^b^*	510.89	0.43
SDR1	0.13*^b^*	0.37	0.42	0.17*^b^*	0.29	0.54	0.20*^b^*	1.05	0.28
SDR2	0.19*^b^*	0.55	0.41	0.20*^b^*	0.49	0.46	0.19*^b^*	0.99	0.27
SDR	0.15*^b^*	0.38	0.44	0.18*^b^*	0.31	0.53	0.19*^b^*	0.94	0.29
AUSNPC	1,912.02*^b^*	4,475.35	0.46	1,696.94*^b^*	3,507.76	0.49	925.27*^b^*	3,844.05	0.32
Grain yield	0.45*^b^*	1.47	0.38	0.37*^b^*	1.61	0.31	1.01*^b^*	1.76	0.53

$H_{a}^{2}$ , broad-sense heritability; $\;{\hat{\sigma }}_{G}^{2}$, genotypic variance; $\;{\hat{\sigma }}_{\varepsilon }^{2}$, error variance; STR8WAP, emerged *Striga* plants 8 weeks after planting (WAP); STR10WAP, emerged *Striga* plants 10 WAP; STR12WAP, emerged *Striga* plants 12 WAP; SDR1 and 2, *Striga* damage rating at 10 and 12 WAP, respectively; SDR, average *Striga* damage rating; AUSNPC, area under *Striga* number progress curve.

^*a*^Significant at *P* < 0.01.

^*b*^Significant at *P* < 0.001.

**Fig. 2.**
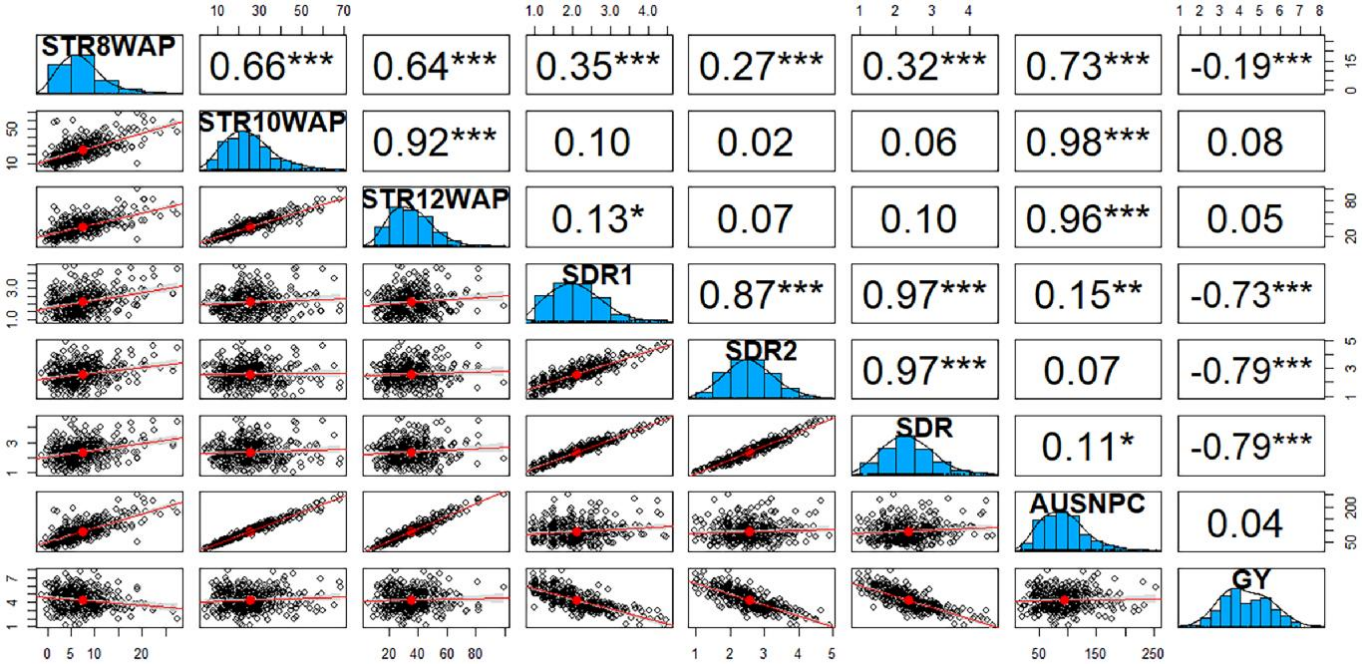
Pearson's correlation coefficients between different *Striga* resistance parameters and grain yield for testcrosses evaluated under artificial *Striga* infestation across 3 test locations in Kenya (Kibos, Alupe, and Siaya) in 2020. STR8WAP, emerged *Striga* plants 8 weeks after planting (WAP); STR10WAP, emerged *Striga* plants 10 WAP; STR12WAP, emerged *Striga* plants 12 WAP; SDR1 and 2, *Striga* damage rating at 10 and 12 WAP, respectively; SDR, average *Striga* damage rating; AUSNPC, area under *Striga* number progress curve; GY, grain yield.

Combined analysis of variance under artificial *Striga* infestation revealed highly significant (*P* < 0.001) variation among hybrids for all traits (Table 2). The G × E interaction was significant for all traits. The ${\hat{\sigma }}_{G}^{2}$ was 3 and 5 times larger than ${\hat{\sigma }}_{GE}^{2}$ for STR10WAP and STR12WAP, respectively. Broad-sense heritability was moderate to high for all *Striga* resistance parameters (0.38–0.65) and grain yield (0.54). The number of emerged *Striga* plants ranged from 4 to 126 with a mean of 8, 27, and 39 at 8, 10, and 12 WAP, respectively. The AUSNPC ranged from 59.5 to 331 m^2^ with a mean of 102.2 m^2^ while grain yield across locations ranged from 3.1 to 6.1 t ha^−1^ with an average of 4.5 t ha^−1^. Significant positive correlation between the 3 *Striga* resistance parameters was revealed (Fig. 2). The correlations between the number of emerged *Striga* plants at 8, 10, and 12 WAP and AUSNPC were high (*r* = 0.73–0.98). *Striga* damage rating showed significant negative correlation with grain yield (*r* = −0.73 to −0.79).

**Table 2.** Summary statistics, variance component estimates, and heritability for different *Striga* resistance parameters and grain yield across 3 locations under artificial *Striga* infestation in 2020.

Trait	Mean	Range	$LSD_{0.05}$	${\hat{\sigma }}_{G}^{2}$	${\hat{\sigma }}_{GE}^{2}$	${\hat{\sigma }}_{\varepsilon }^{2}$	$H_{b}^{2}$
STR8WAP	8	4–32	6.3	9.24*^c^*	9.94*^c^*	72.39	0.38
STR10WAP	27	16–82	14.1	80.02*^c^*	22.65*^b^*	322.99	0.57
STR12WAP	39	21–126	18.4	181.61*^c^*	32.99*^a^*	520.38	0.65
SDR1	2.1	1.5–3.9	0.6	0.12*^c^*	0.05*^c^*	0.57	0.51
SDR2	2.6	1.8–4.4	0.6	0.13*^c^*	0.06*^c^*	0.68	0.49
SDR	2.3	1.6–4.2	0.5	0.11*^c^*	0.05*^c^*	0.55	0.51
AUSNPC	102.2	59.5–331.0	50.0	1,182.87*^c^*	295.5*^b^*	3,966.04	0.61
Grain yield	4.5	3.1–6.1	1.0	0.40*^c^*	0.22*^c^*	1.61	0.54

$H_{b}^{2}$ , broad-sense heritability; ${\hat{\sigma }}_{e}^{2}$, error variance; ${\hat{\sigma }}_{G}^{2}$, genotypic variance; ${\hat{\sigma }}_{GE}^{2}$, genotype by environmental variance; STR8WAP, emerged *Striga* plants 8 weeks after planting (WAP); STR10WAP, emerged *Striga* plants 10 WAP; STR12WAP, emerged *Striga* plants 12 WAP; SDR1 and 2, *Striga* damage rating at 10 and 12 WAP, respectively; SDR, average *Striga* damage rating; AUSNPC, area under *Striga* number progress curve.

^*a*^Significant at *P* < 0.05.

^*b*^Significant at *P* < 0.01.

^*c*^Significant at *P* < 0.001.

### Prediction accuracy

The 606 DH lines were genotyped with 8,439 markers of which 5,380 high quality rAmpSeq markers were used for the analysis. Three cross-validation (CV) schemes were used to assess the prediction accuracy of the reaction norm model. The CV0 and CV2 were used to determine the prediction accuracy of the model when estimating the performance of previously phenotyped lines in new environments while CV1 was applied when assessing the accuracy of the model when estimating the performance of newly developed lines that have not been tested before. The results indicate moderate prediction accuracies for most traits at Kibos and Alupe (Table 3). For individual locations, Alupe showed better prediction accuracies for most traits across the 3 CV schemes while Siaya had the lowest prediction accuracies for most *Striga* resistance parameters. The prediction accuracies for grain yield were equal for the three locations for CV0 (0.59) and similar for Alupe and Siaya for CV2. For across location analysis, the predictive accuracy of the model was better for CV0 compared to both CV2 and CV1 for most traits except number of emerged *Striga* plants at 10 and 12 WAP (Table 3). Overall, the prediction accuracy of CV0 (0.24–0.59) and CV2 (0.20–0.56) was higher than that of CV1 (0.05–0.29). Grain yield generally showed better prediction accuracies (CV0 and CV2) across the trial locations compared to the *Striga* resistance parameters.

**Table 3.** Prediction accuracies for *Striga* resistance parameters and grain yield using 3 cross-validation schemes (CV0, CV1, and CV2) for Kibos, Alupe, and Siaya and across locations under artificial *Striga* infestation.

Trait	CV0				CV1				CV2			
	Kibos	Alupe	Siaya	Across locations (weighted *r*)	Kibos	Alupe	Siaya	Across locations (weighted *r*)	Kibos	Alupe	Siaya	Across locations (weighted *r*)
STR8WAP	0.39	0.43	0.07	0.30	0.35	0.15	0.07	0.19	0.34	0.18	0.08	0.20
STR10WAP	0.37	0.40	0.24	0.34	0.33	0.46	0.10	0.29	0.36	0.56	0.19	0.37
STR12WAP	0.26	0.17	0.30	0.24	0.31	0.43	0.19	0.31	0.31	0.53	0.26	0.37
SDR1	0.29	0.29	0.28	0.29	0.06	0.10	0.00	0.05	0.31	0.28	0.18	0.26
SDR2	0.64	0.59	0.36	0.53	0.01	0.10	0.20	0.10	0.27	0.36	0.35	0.33
SDR	0.35	0.36	0.33	0.35	0.01	0.04	0.13	0.06	0.27	0.28	0.30	0.29
AUSNPC	0.40	0.53	0.25	0.39	0.34	0.43	0.10	0.29	0.38	0.56	0.21	0.38
Grain yield	0.59	0.59	0.59	0.59	0.26	0.30	0.20	0.25	0.63	0.53	0.52	0.56

CV0, cross-validation 0; CV1, cross-validation 1; CV2, cross-validation 2; STR8WAP, emerged *Striga* plants 8 weeks after planting (WAP); STR10WAP, emerged *Striga* plants 10 WAP; STR12WAP, emerged *Striga* plants 12 WAP; SDR1 and 2, *Striga* damage rating at 10 and 12 WAP, respectively; SDR, average *Striga* damage rating; AUSNPC, area under *Striga* number progress curve.

### Genomic estimated breeding values

**Fig. 3.**
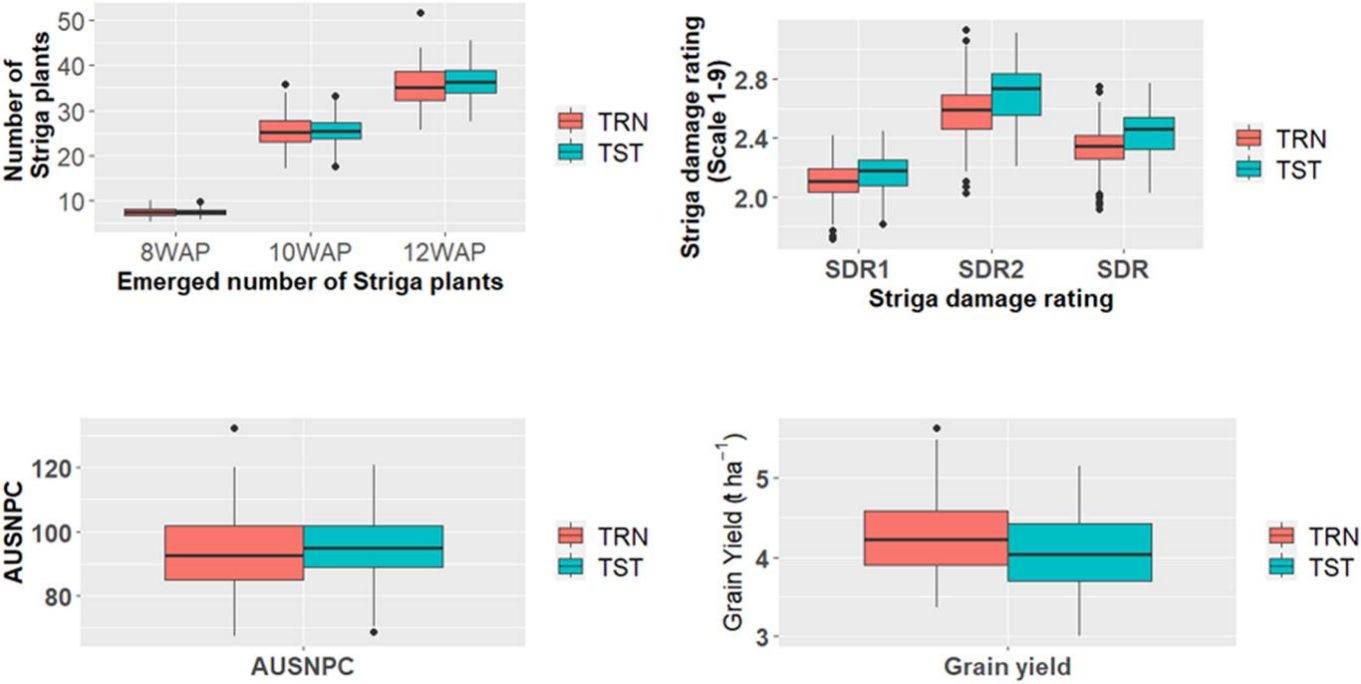
Boxplots of mean GEBVs for *Striga* resistance parameters and grain yield for the training (TRN) and testing (TST) sets across the trial locations. 8WAP, emerged *Striga* plants 8 weeks after planting (WAP); 10WAP, emerged *Striga* plants 10 WAP; 12WAP, emerged *Striga* plants 12 WAP; SDR1 and 2, *Striga* damage rating at 10 and 12 WAP, respectively; SDR, average *Striga* damage rating; AUSNPC, area under *Striga* number progress curve.

**Fig. 4.**
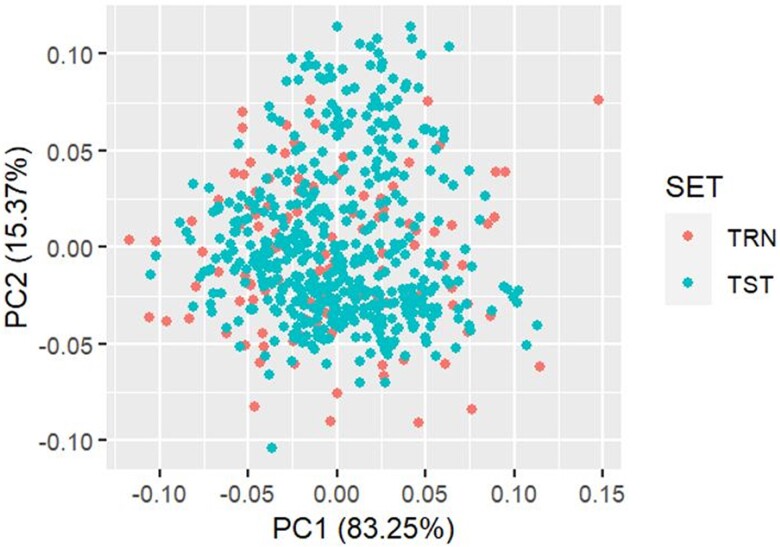
Principal component analysis of the GEBVs for the TRN and TST sets. The x- and y-axes are the first and the second principal components, respectively. TRN, training population; TST, testing population.

The GEBVs of the lines in the testing set (TST) were computed from both marker and phenotypic data (BLUEs) of the training set (TRN) using the reaction norm model. The mean GEBVs of *Striga* resistance parameters and grain yield for both the TRN and TST sets across the 3 trial locations are presented in Fig. 3, and their distribution in Supplementary Fig. 1. The results indicated that there was a close relationship between the GEBVs in TRN and TST sets (Fig. 4). The mean GEBVs were either equal in the TRN and TST sets for STR8WAP and STR10WAP or slightly higher in the TST compared to the TRN for the other traits except grain yield for which the mean of the TST (4.0 t ha^−1^) was lower than that of the TRN (4.26 t ha^−1^). The mean GEBV of emerged *Striga* plants ranged from 7.5 for STR8WAP to 35.6 for STR12WAP in the TRN and 7.5 for STR8WAP to 36.4 for STR12WAP in the TST sets (Fig. 3). Results showed that 45, 61, and 63 lines in the TRN had lower GEBVs for STR8WAP, STR10WAP, and STR12WAP, respectively. On the other hand, about 50% of the lines in the TST set had lower emerged *Striga* plants in comparison with the mean at STR8WAP, STR10WAP, and STR12WAP. The mean GEBV for *Striga* damage was 2.1 and 2.6 for SDR1 and SDR2, respectively, in the TRN, while that of the TST was 2.2 (SDR1) and 2.7 (SDR2) (Fig. 3). The predicted GEBV of SDR ranged from 1.7 (SDR1) to −3.1 (SDR2) for the TRN and 1.8 (SDR1) to −3.1(SDR2) in the TST. A total of 27 and 144 DH lines showed lower GEBVs for SDR than the mean for the TRN and TST, respectively. In total, 56% (TRN) and 48.4% (TST) of the lines showed smaller AUSNPC than the mean GEBV. Additionally, 50 and 239 lines had higher predicted GY than the mean in the TRN and TST sets, respectively. Of the 606 DH lines, 282, 307, and 313 lines had a lower number of emerged *Striga* plants than the mean GEBVs at 8, 10, and 12 WAP, respectively.

## Discussion

Breeding for *Striga* resistance in maize presents a unique challenge owing to the quantitative nature of *Striga* inheritance, narrow genetic base of elite *Striga* resistant germplasm, constrained phenotyping capacity, and high phenotyping costs. Breeding for *Striga* resistance therefore requires multiple approaches including classical breeding, use of molecular markers, and a combination of the 2 approaches to address these challenges. Our objectives were to assess the prediction accuracy of genomic selection in determining the genetic values of tested and untested DH lines under artificial *Striga* infestation.

### Phenotypic variation and heritability

The testcrosses in this study were developed from a diverse set of DH lines whose pedigree included *Striga*-susceptible but elite mid-altitude tropical maize lines from CIMMYT and *Striga* resistant donor lines from IITA. The results indicated significant genotype and G × E interaction for all traits possibly due to differential responses to *Striga* infestation among testcrosses arising from the diverse genetic backgrounds of the lines and differences among the locations used. The differences at the locations could be attributed to climatic and edaphic factors (Menkir *et al.* 2012; Makumbi *et al.* 2015). The genetic variance was 9 and 20 times larger at 10 WAP and 12 WAP, respectively, than at 8 WAP which corroborates with results from an earlier study (Gowda *et al.* 2021). This suggests that there is sufficient variability among these hybrids for *Striga* emergence that can be uncovered at 10 and 12 WAP and to reduce phenotyping costs at 8 WAP. The genetic variance recorded in this study was larger than G × E variance, similar to the result reported by Menkir and Kling (2007) and Gowda *et al*. (2021). The observed large genetic variance could arise from the use of lines containing *Striga* resistant alleles of diverse origins (Menkir 2011; Menkir *et al.* 2012) and diverse elite mid-altitude lines from CIMMYT. Furthermore, use of DH populations could have contributed to the observed larger genetic variance (Gallais 1990).

The variability observed between the number of emerged *Striga* plants and *Striga* damage rating among locations suggests the likelihood of different *Striga* ecotypes exhibiting variable virulence as well as the effects of different climatic and edaphic factors. Mbuvi *et al*. (2017) reported significant variability among *Striga* ecotypes at Kibos and Alupe with the ecotypes at Kibos found to be more virulent on sorghum compared to the ecotypes at Alupe. This may explain the low *Striga* damage rating observed at Alupe despite the high number of emerged *Striga* plants recorded at this site. Heritability estimates for most of the *Striga* resistance parameters and grain yield across locations were moderate, suggesting that selection of superior inbred lines with relevant *Striga* resistance traits should be possible. Heritability estimates for *Striga* resistance parameters like emerged *Striga* counts have been variable in several studies, ranging from moderate (Adewale *et al.* 2020; Gowda *et al.* 2021; Okunlola *et al.* 2023) to high (Menkir *et al.* 2012) based on differences in the germplasm used.

The correlation between the number of emerged *Striga* plants at 10 and 12 WAP and grain yield was low and nonsignificant. This corroborates the findings by Adewale *et al*. (2020), Stanley *et al*. (2021), and Okunlola *et al*. (2023) but is contrary to results by Menkir and Kling (2007) and Gowda *et al*. (2021). On the other hand, SDR showed significant negative correlations with grain yield, suggesting that SDR is a useful parameter for measuring *Striga* resistance under artificially infested conditions and could be used to select inbred lines combining lower *Striga* damage and higher grain yield. Correlations between 2 traits may be due to pleiotropy, linkage, or both, amount of linkage disequilibrium, and the effect of the environment. The low correlation between grain yield and number of emerged *Striga* plants at 10 and 12 WAP suggests a lack of linkage between genes controlling these traits. Parents of the inbred lines used in the present study show significant negative correlation between SDR and STR, and between grain yield and SDR, and STR under *Striga* infestation. It is possible that the lines derived from crosses between IITA and CIMMYT lines may not carry all the favorable alleles derived from the parental lines leading to weak correlation among these traits. Selection-induced changes can modify the genetic correlation between traits either by altering the pattern of polymorphism at loci with pleiotropic effects or by changing the linkage disequilibrium among closely linked loci (Lande 1984). While these correlations are useful, more detailed investigations should focus on genetic correlations between various *Striga* resistance parameters and grain yield based on a larger data set (multiple environments and seasons), as these provide the breeder with a better understanding of the relationship among traits (pleiotropy or linkage) and could have implications for application of indirect selection in a breeding program.

### Genomic prediction

Genotype × environment interactions significantly influence phenotypic performance and ultimate selection potential in crops (Des Marais *et al.* 2013). We used the reaction norm model which considers the epistatic effects resulting from various interactions among genotypes, markers, and the environment to estimate an individual's phenotype or its performance in new environments (Jarquín *et al.* 2014). Prediction of genetic values of lines in environments in which they were not tested (CV0 and CV2) resulted in low to moderate prediction accuracy. This suggests that estimation of the GEBVs of lines in new environments is possible for *Striga* resistance parameters and grain yield. This kind of genetic value prediction is akin to sparse testing due to the use of information on the performance of lines in correlated environments (Burgueño *et al*. 2012; Mageto *et al.* 2020). This is attributed to the ability of the reaction norm model to leverage information from relatives resulting from the interaction of genotypes within and across environments and correlated environments (Burgueño *et al.* 2012). The prediction accuracy for CV0, CV1, and CV2 for *Striga* resistance parameters obtained in this study was lower than that reported by Gowda *et al*. (2021). However, our results indicate 14–19% better prediction accuracy for grain yield compared to Gowda *et al*. (2021) for the 3 CV schemes. These differences in results may be due to the complexity of *Striga* resistance, besides the differences in germplasm and prediction models used. The prediction accuracy was relatively low with the application of GS to newly developed lines (CV1). A similar finding was reported by Gowda *et al*. (2021) for *Striga* resistance in maize and by Semagn *et al*. (2022) for multiple disease resistance in wheat. The low prediction accuracy with CV1 is attributed to its reliance on the phenotypic values and genetic relationships of other lines (Burgueño *et al.* 2012; Mageto *et al.* 2020).

The predictive power of genetic models is significantly affected by low trait heritability (Liu *et al.* 2018). The relatively low to moderate prediction accuracy observed for *Striga* resistance parameters in this study was possibly due to the low trait heritability and relatively small training population size (Heffner *et al.* 2011; Ornella *et al.* 2012). The moderate heritability for most traits may partly explain the low to moderate prediction accuracies recorded for *Striga* resistance parameters in this study. A positive correlation between high trait heritability and high prediction accuracy was reported for kernel zinc concentration in maize (Mageto *et al.* 2020). The limited TRN size was due to the limited area available for artificial *Striga* screening, which in turn limited the number of testcrosses that could be evaluated in the field. A large TRN set is important for increased prediction accuracy (Lorenz *et al.* 2012; Gowda *et al.* 2015; Beyene *et al.* 2019). However, the level of prediction accuracy achieved in this study should still allow for application of GS by removing lines with the least favorable GEBVs for key *Striga* resistance traits before testcrossing (Edriss *et al.* 2017). The moderate prediction accuracies for some traits could be attributed to the close relationship between the TRN and TST sets as well as the model used (Jarquín *et al.* 2017; Brandariz and Bernardo 2019). In this study, we identified 300 lines with desirable GEBVs for fewer emerged *Striga* plants at 10 and 12 WAP. These lines putatively have good alleles that could reduce *Striga* emergence in maize. These lines should be tested in hybrid combinations under artificial *Striga* infestation and optimal conditions to identify the most suitable lines combining *Striga* resistance and other adaptive traits. Selection of genotypes that support a reduced number of emerged *Striga* plants should help in curtailing the replenishment of the *Striga* seed bank in the soil.

### Prospects in breeding for resistance to *Striga*

Breeding for *Striga* resistance is one of the strategies that can be used to increase maize grain yield while also contributing to reduced *Striga* seed bank in the soil in *Striga* affected regions in SSA. Maize breeding programs targeting *Striga* resistance are faced with a multitude of challenges which could be overcome by a combination of conventional and molecular technologies. With advances in genomic approaches and lower genotyping costs, the integration of classical and genomic-assisted breeding strategies has the potential to address some of the limitations of breeding for *Striga* resistance to enhance genetic gains. The application of genomic selection for the improvement of complex traits in tropical maize has been documented (Crossa *et al.* 2010; Vivek *et al.* 2017; Beyene *et al.* 2019, 2021). The application of DH technology for efficient inbred line development (Prasanna *et al.* 2012; Chaikam *et al.* 2019) could be used to unravel larger genetic variability for selection efficiency. The application of forward breeding for key diseases such as maize lethal necrosis (MLN) and maize streak virus (MSV) for new DH lines should reduce the number of DH lines to be phenotyped under artificial *Striga* infestation and hence reduce phenotyping costs (Prasanna *et al.* 2021).

Our results show that there is potential to implement GS in breeding for *Striga* resistance in maize. The application of GS in breeding for *Striga* resistance should be integrated with the use of DH lines, and application of sparse phenotyping. Sparse testing has been reported to improve the efficiency of GS through optimal resource utilization and enhancement of prediction accuracy (Jarquín *et al.* 2020; Montesinos-López *et al.* 2023b). The use of sparse testing and GS in selection for target traits has been reported in wheat and maize (Jarquín *et al.* 2020; Atanda *et al.* 2022). The application of sparse testing and GS in breeding for *Striga* resistance requires optimization of the TRN set. Montesinos-López *et al*. (2023a) suggested that the optimization of TRN populations in GS can be enhanced through appropriate prediction models and experimental designs in sparse testing. Therefore, detailed investigations on TRN size under *Striga* infestation may be necessary before scaling the application of GS in maize *Striga* resistance breeding programs. By leveraging genomic relationships and tapping into hidden replicated alleles, genomic prediction offers the benefits of more accurate predictions and effective reduction of the high costs associated with phenotyping of large sets of individuals (Vivek *et al.* 2017; Wang *et al.* 2020). Integration of several genomics-enabled techniques including use of environmental data (Jarquín *et al.* 2014; Jarquín *et al.* 2020; Crossa *et al.* 2022) should assist in achieving better genetic gains for reduced *Striga* infestation and higher grain yield under *Striga* infestation. While the application of modern breeding techniques can lead to higher genetic gains in breeding for *Striga* resistance, part of the solution to the problem of *Striga* in Africa will be integrated *Striga* management that encompasses multiple control strategies to obtain maize yield sustainability. Stacking multiple stress tolerance in addition to *Striga* tolerance (e.g. Menkir *et al.* 2020) should improve maize productivity in the *Striga* affected agroecologies in SSA.

## Conclusions

Genomic-enabled selection can be an important tool in improving the efficiency of breeding for *Striga* resistance in maize. Using the reaction norm model with 2 cross-validation schemes (CV0 and CV2), our findings reveal moderate prediction accuracies for 3 key *Striga* resistance traits (STR10WAP, STR12WAP, and AUSNPC) and grain yield (GY) at 2 out of the 3 locations under artificial *Striga* infestation. The reaction norm model sufficiently modeled the interactions among genotypes, environments, markers, and G × E effects, to obtain accurate GEBVs. This study revealed a close relationship between the GEBVs across the TRN and TST sets for key *Striga* resistance traits, with 300 DH inbred lines displaying favorable GEBVs for these parameters. These results suggest that application of genomic-enabled strategies can facilitate improvements in *Striga* resistance in maize. These results provide a foundational framework for the potential integration of GS in breeding for *Striga* resistance in maize across sub-Saharan Africa. Future research should focus on optimizing the training population size for large-scale application of GS and testing a combination of GS and sparse phenotyping approaches in field evaluation of lines and hybrids for resistance to *Striga* under artificial infestation conditions.

## Supplementary Material

jkae186_Supplementary_Data

## Data Availability

Supplementary data are available. Supplementary Table 1—Pedigrees of DH lines in GS study gives the list and pedigrees of DH lines used in the study. Supplementary Fig. 1 shows the distribution of the GEBVs for the number of emerged *Striga* plants for the training and testing populations. The phenotypic and marker data are freely available from CIMMYT's Dataverse (https://hdl.handle.net/11529/10549033). File named Phenotypic_Data.CSV contains phenotypic data from 232 testcross (TC) hybrids. File named GS_Marker_Data.CSV contains genotypic data for 606 doubled haploid (DH) lines. Supplemental material available at G3 online.
