# Supplementary figures and images for: Genomic prediction of the performance of tropical doubled haploid maize lines under artificial Striga hermonthica (Del.) Benth. infestation
Source: G3 (Bethesda). 2024 Aug 12;14(10):jkae186. doi: 10.1093/g3journal/jkae186 (PMC11457060; doi:10.1093/g3journal/jkae186)

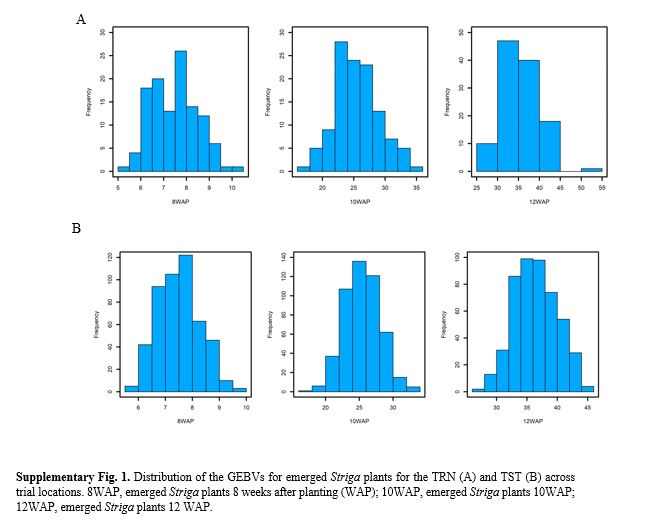

Supplement: jkae186_Supplementary_Data [file jkae186_supplementary_data.zip › Supplementary_Figure_1_G3-2024-405125.png]
